# Solar cycle response and long‐term trends in the mesospheric metal layers

**DOI:** 10.1002/2016JA022522

**Published:** 2016-07-27

**Authors:** E. C. M. Dawkins, J. M. C. Plane, M. P. Chipperfield, W. Feng, D. R. Marsh, J. Höffner, D. Janches

**Affiliations:** ^1^ School of Chemistry University of Leeds Leeds UK; ^2^ National Center for Atmospheric Science, School of Earth and Environment University of Leeds Leeds UK; ^3^ NASA Goddard Space Flight Center Greenbelt Maryland USA; ^4^ Department of Physics Catholic University of America Washington District of Columbia USA; ^5^ National Center for Atmospheric Research Boulder Colorado USA; ^6^ Leibniz‐Institute for Atmospheric Physics Kühlungsborn Germany

**Keywords:** mesospheric metal, solar cycle, long‐term trends, modeling, satellite retrieval

## Abstract

The meteoric metal layers (Na, Fe, and K)—which form as a result of the ablation of incoming meteors—act as unique tracers for chemical and dynamical processes that occur within the upper mesosphere/lower thermosphere region. In this work, we examine whether these metal layers are sensitive indicators of decadal long‐term changes within the upper atmosphere. Output from a whole‐atmosphere climate model is used to assess the response of the Na, K, and Fe layers across a 50 year period (1955–2005). At short timescales, the K layer has previously been shown to exhibit a very different seasonal behavior compared to the other metals. Here we show that this unusual behavior is also exhibited at longer timescales (both the ~11 year solar cycle and 50 year periods), where K displays a much more pronounced response to atmospheric temperature changes than either Na or Fe. The contrasting solar cycle behavior of the K and Na layers predicted by the model is confirmed using satellite and lidar observations for the period 2004–2013.

## Key Points

- First study of response of modeled metal layers to changing atmosphere
- K is the only metal to exhibit pronounced response to atmospheric changes at 11 year solar cycle and longer‐term (50 year) timescales
- K layer may be good candidate as a sensitive indicator of long‐term changes in the mesosphere/lower thermosphere region

## 1 Introduction

There has been increasing interest in the middle and upper atmospheric response to anthropogenic climate change ever since the model prediction of *Roble and Dickinson* [1989] which demonstrated that the global average mesospheric temperature would cool by approximately 7–12 K at altitudes below 100 km for a doubled‐CO_2_ scenario. Since then, modeling studies have helped to identify the main drivers for past and current observed trends, most notably the increase of greenhouse gases [e.g., *Akmaev et al*., 2006; *Garcia et al*., 2007], which act as radiative coolers at these altitudes, in contrast to their radiative warming properties within the lower atmosphere. Other identified drivers include changes in O_3_ [e.g., *Akmaev et al*., 2006], changes in atmospheric dynamics [e.g., *Jacobi*, 2014], and changes in both solar and geomagnetic activity [e.g., *Mikhailov*, 2002; *Schmidt et al*., 2006; *Marsh et al*., 2007]. A number of reviews on these trends have been published in recent years, including *Beig* [2011], *Laštovička* [2013], and *Plane et al*. [2015].

Compared to the lower atmosphere, the upper mesosphere/lower thermosphere region (MLT, 75–110 km) is relatively poorly understood due to a critical lack of observations. However, the MLT region is very sensitive to perturbations and is subject both to the effects of solar irradiation, the solar wind, and ionospheric impacts from above, and to the influences of upward propagating atmospheric waves and dynamical forcing from lower altitudes [e.g., see *Plane*, 2003]. The response of the MLT to both the 11 year solar cycle and longer‐term changes are not well known. Issues with data continuity, differences in spatiotemporal sampling, the choice of trend analysis employed, and the uncertainties associated with each measurement technique mean that trend studies are difficult and highly uncertain [e.g., see *Remsberg*, 2007, 2008; *Offermann et al*., 2010; *Beig*, 2011; *Lübken et al*., 2013; *Forbes et al*., 2014]. Within the MLT region, these considerations are particularly important due to the relatively limited number of observations available and the need to detect weak trends in what is typically geophysically variable data.

Metal layers, which form as a result of meteoric ablation [e.g., see *Plane*, 2003], exist as a layer of neutral atoms between approximately 80 and 105 km altitude, and their abundance is dependent on complex interactions between chemical reactions and dynamics. Above 105 km, the metals persist mostly as metal ions. Below 80 km, these metals form various compounds (e.g., carbonates, hydroxides, and oxides). These compounds polymerize into nanometer‐sized meteoric smoke particles which likely act as cloud condensation nuclei through the mesosphere and stratosphere, before being deposited at the Earth's surface approximately 4 years later [*Dhomse et al*., 2013]. The spatiotemporal concentrations of these metals are dependent on both chemistry and dynamics; the observation and accurate modeling of such neutral metal layers permit an increased understanding of such processes affecting the MLT region [*Feng et al*., 2013; *Marsh et al*., 2013; *Plane et al*., 2014, 2015; *Langowski et al*., 2015].

Much of the study of the metal layers to date is as a result of lidar observations [e.g., *Megie and Blamont*, 1977; *Eska et al*., 1998, 1999; *Gerding et al*., 2000; *Gardner et al*., 2005; *Höffner and Lübken*, 2007; *Chu et al*., 2011], laboratory measurements [e.g., see *Cox and Plane*, 1997], and satellite observations [e.g., *Fussen et al*., 2004, 2010; *Fan et al*., 2007; *Gumbel et al*., 2007; *Hedin and Gumbel*, 2011; *Dawkins et al*., 2014; *Langowski et al*., 2014, 2015]. Such studies have revealed that notable differences exist between the metals in terms of their atmospheric abundance and seasonal variation [e.g., see *Gerding et al*., 2000; *Plane*, 2003; *Plane et al*., 2014]. Whole‐atmosphere modeling with the National Center for Atmospheric Research (NCAR) Whole Atmosphere Community Climate Model (WACCM) and the input of metal chemistry modules (for Na, K, Fe, and Mg) offer an excellent opportunity to test our understanding of this poorly understood region via comparison with available measurements.

The aims of this work are twofold: to explore the comparative responses of individual metal species to both the ~11 year solar cycle and longer‐term changes and to assess whether any of these metals act as sensitive indicators of climate change. In section 2, the suitability of using WACCM as a tool to study the long‐term response of the Na, Fe, and K metal layers, and the resulting predictions of the model over a 50 year period, are examined. In section 3, the modeled responses of the Na and K layers to the solar cycle are compared with observations between 2004 and 2013. The summary and conclusions are then presented in section 4.

## 2 Long‐Term Trends in the Metal Layers

### 2.1 The Use of WACCM for Long‐Term Trend Studies Within the MLT Region

NCAR WACCM is a comprehensive coupled chemistry‐climate model and is part of the Community Earth System Model framework. The standard model extends from the Earth's surface up to ~140 km, with a horizontal resolution of 1.9° latitude × 2.5° longitude and 66 vertical levels with a vertical resolution of approximately 1.5 km in the lower atmosphere and 3.5 km in the MLT region. WACCM consists of a fully interactive chemistry scheme and includes shortwave heating and photolysis from the visible to extreme UV [*Marsh et al*., 2007]. Above 60 km, it incorporates non‐LTE (local thermal equilibrium) IR transfer. It includes a parameterization for gravity waves from convection and fronts (and their subsequent breaking within the mesopause region) and includes thermospheric processes such as aurora, ion chemistry, and molecular diffusion. Metal chemistry modules have been added for Na [*Marsh et al*., 2013], Fe [*Feng et al*., 2013], Mg [*Langowski et al*., 2015], and K [*Plane et al*., 2014], and these studies demonstrate that WACCM is able to adequately simulate the seasonal distribution of these metal layers.

In this section, output from a free‐running version of NCAR WACCM is employed to investigate long‐term trends in the Na, Fe, and K metal layers over a 50 year period (1955–2005). The employed standard WACCM model component set uses the prescribed sea surface temperatures and ocean and ice coverage and interactive community land model with daily solar data and solar proton events [*Marsh et al*., 2013]. The longer‐term changes in these metal layers are examined in context relative to the changes in temperature and other minor chemical constituents relevant to the metal chemistry. Key questions to be addressed include the following: Do the metal layers display different long‐term trends in column density compared to one another? What are the driving factors for the behavior? Are there changes in the metal layer characteristics, such as the centroid altitude and root‐mean‐square (RMS) width, and is this consistent with the observed and modeled changes in the MLT region?

**Figure 1:**
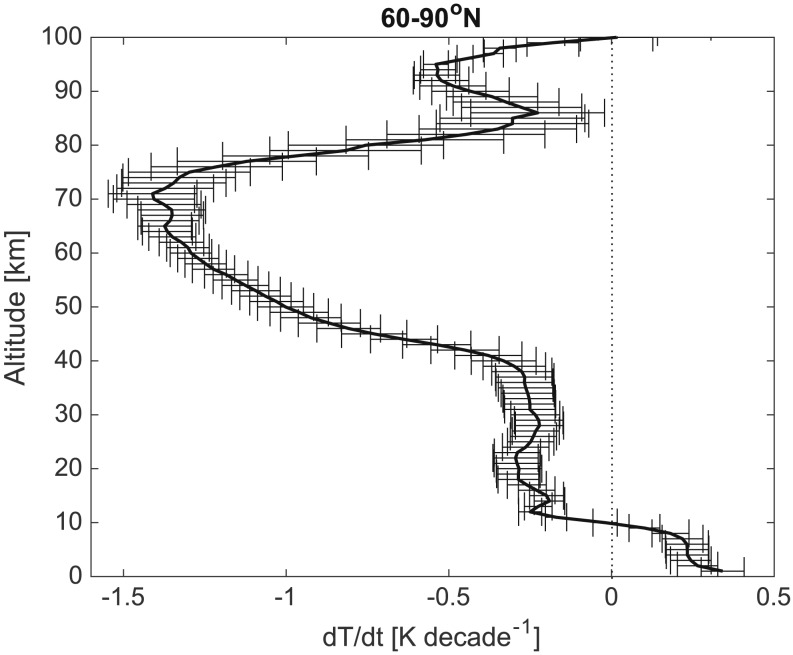
Average decadal linear trends in temperature as simulated by WACCM for 60–90°N, across the period 1955–2005 (units: K decade^−1^). The horizontal error bars represent the associated mean standard error of the trend.

Previous work by *Garcia et al*. [2007] has shown that WACCM is capable of adequately simulating the observed long‐term trends in both temperature and O_3_. Decadal temperature trends are determined using a linear regression of the annual means, and an example high‐latitude (60–90°N) WACCM‐simulated decadal temperature trend (across the 1955–2005 period) is presented in Figure 1. A warming trend occurs within the troposphere, with a maximum warming of approximately +0.22 K decade^−1^ below 5 km altitude (mean temperature change of +0.19 K decade^−1^ between 0 and 10 km). In the stratosphere above the tropopause region, cooling trends dominate with a maximum rate of ~ −0.9 K decade^−1^ at ~45–50 km, which is associated with the long‐term changes in the stratospheric O_3_ layer. Cooling trends of between −0.17 and −0.69 K decade^−1^ (mean: −0.34 K decade^−1^) are simulated within the mesopause region (80–90 km). These cooling trends above 20 km are considerably larger than the tropospheric warming trend.

### 2.2 Detecting Long‐Term Linear Trends in the Modeled Metal Layers

In order to detect long‐term linear trends in a data set (e.g., over a 50 year period), other sources of variation within the data must first be removed. The dominant sources include both the annual cycle monthly variation and any variation associated with the solar cycle. The seasonal variations are removed by considering only annual means.

To determine the linear trend and solar cycle response in either temperature or a constituent, we use a multilinear regression fit to the time series of annual means of the following form:

$$y\left(t\right)=a+bt+cS\left(t\right)$$

where *y*(*t*) is the data time series; *S*(*t*) is the solar irradiance, which is approximated by the annual mean of the 10.7 cm flux (*F*_10.7_); and *t* is the time in years since 1955. Coefficient *a* is the value of the fit at 1955, *b* is the linear trend term (per year), and *c* is the solar coefficient reported in units of 100 solar flux units (100 sfu; 1 sfu = 10^−22^ W m^−2^ Hz^−1^).

**Figure 2:**
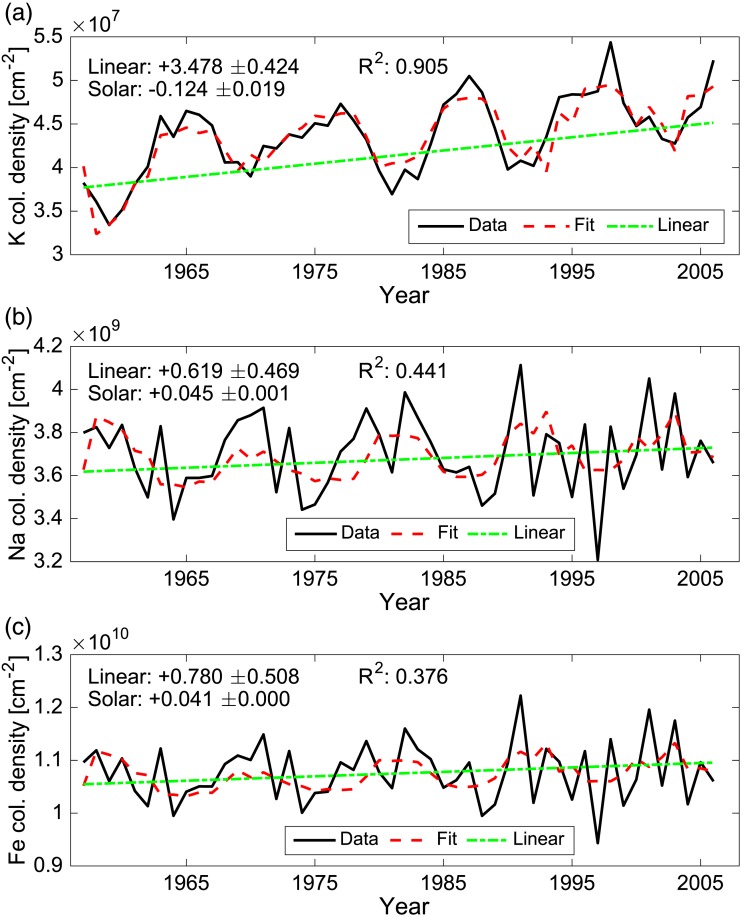
Example fitting routine for (a) K, (b) Na, and (c) Fe. The original metal annual mean column time series is shown as a solid black line (units: atoms cm^−2^). The multilinear regression model is shown as the red dashed line. The linear component of the fitted model is shown in a green line. For each metal, the linear and solar contributions to the fit are reported (along with their associated error), and the *R* ^2^ and *p* value. The linear trend is reported as the percent change in column density per decade, while the solar trend is reported as the percent change per 100 sfu.

Figure 2a provides an example of a multilinear regression fit to the WACCM K annual zonal mean column density time series (1955–2005) for 60–90°N. The linear and solar trend components, their associated errors, and the *R* ^2^ term of the multilinear regression model are reported; within this latitude range, the K column density is increasing at a rate of approximately + (3.48 ± 0.42)% decade^−1^, with a solar signal of −(0.12 ± 0.02)%/100 sfu. The *R* ^2^ term has a value of +0.905 (*p* < 0.01) indicating that the multilinear regression model generally represents the original WACCM data well.

The same technique is also used for WACCM‐modeled output for Na and Fe (also shown in Figure 2). In these cases, the least squares expression does not adequately capture the temporal variation seen in the annual WACCM data. A fast Fourier transform (FFT) spectral analysis was performed for each of the example (60–90°N) plots to analyze whether there are any hidden variations in the original monthly time series not accounted for within the Na and Fe data, which would result in an improved multilinear model fit. The results for these FFTs are presented in the supporting information (Figure S1). For each metal, there is a dominant annual periodicity component. However, while K shows an additional frequency which corresponds approximately to the ~11 year solar cycle response, neither Na nor Fe shows any other notable underlying dominant frequencies. Thus, no further improvement or refinement of the multilinear regression model was made.

### 2.3 The 50 Year Long‐Term Trends in the Modeled K, Na, and Fe Metal Layers

Long‐term trends within the WACCM‐simulated K, Na, and Fe metal layers were analyzed as a function of zonal mean latitude band (binned into 30° bands) across the time period 1955–2005. Each data set is normalized by its respective multiyear mean in order to facilitate an easier comparison. A summary of the linear trends for each metal as a function of latitude is provided in Table 1. K is the only metal which exhibits a clear increasing trend in column density at all latitudes with *R* ^2^ values typically exceeding +0.89 (all with *p* values <0.01). In contrast, both Na and Fe generally show near‐zero or very weak trends that vary with latitude with *R* ^2^ values that are considerably lower (ranging at statistically significant values of +0.309 < *R* ^2^ < +0.441 (full range: +0.079 < *R* ^2^ < +0.441) for Na and +0.376 < *R* ^2^ < +0.564 (full range: +0.249 < *R* ^2^ < +0.622) for Fe). Taking into account only those results that are statistically significant, the K linear trends typically range between a factor 3 and 36 times larger in magnitude than those of Na and Fe, varying with latitude. Overall, the results indicate that the K column densities have increased by approximately +15–17% depending on latitude across the 50 year period, compared to the Na and Fe layers where trends vary between −5% and +4%.

**Table 1:** Trends in the WACCM Metal Layers as a Function of Latitude Band (90°N to 90°S, in 30° Intervals)^a^

	Linear and 11 Year Solar Cycle Trends in WACCM Metal Layers (Linear Units: % Change Decade^−1^, Solar Trend Units: % (100 sfu)^−1^)		
Latitude Band	K	Na	Fe
60–90°N	+3.478 ± 0.424	+0.619 ± 0.469	+0.780 ± 0.508
	−0.124 ± 0.019	+0.045 ± 0.001	+0.041 ± 0.000
	*R* ^2^ = 0.905 (*p* < 0.01)	*R* ^2^ = 0.441 (*p* < 0.01)	*R* ^2^ = 0.376 (*p* < 0.01)
30–60°N	+3.239 ± 0.424	+0.725 ± 0.338	+0.878 ± 0.359
	−0.133 ± 0.016	+0.022 ± 0.001	−0.001 ± 0.013
	*R* ^2^ = 0.907 (*p* < 0.01)	*R* ^2^ = 0.326 (*p* < 0.05)	*R* ^2^ = 0.249 (*p* = 0.081)
0–30°N	+3.055 ± 0.453	+0.410 ± 0.320	+0.416 ± 0.401
	−0.136 ± 0.015	−0.001 ± 0.028	−0.035 ± 0.000
	*R* ^2^ = 0.896 (*p* < 0.01)	*R* ^2^ = 0.079 (*p* = 0.587)	*R* ^2^ = 0.515 (*p* < 0.01)
0–30°S	+3.071 ± 0.455	+0.177 ± 0.312	+0.219 ± 0.384
	−0.144 ± 0.013	−0.013 ± 0.002	−0.047 ± 0.000
	*R* ^2^ = 0.902 (*p* < 0.01)	*R* ^2^ = 0.309 (*p* < 0.05)	*R* ^2^ = 0.622 (*p* < 0.01)
30–60°S	+3.064 ± 0.500	−0.173 ± 0.211	−0.086 ± 0.230
	−0.148 ± 0.012	−0.008 ± 0.003	−0.027 ± 0.000
	*R* ^2^ = 0.888 (*p* < 0.01)	*R* ^2^ = 0.333 (*p* < 0.05)	*R* ^2^ = 0.578 (*p* < 0.01)
60–90°S	+2.993 ± 0.698	−1.055 ± 0.504	−0.914 ± 0.634
	−0.162 ± 0.011	+0.019 ± 0.001	+0.015 ± 0.000
	*R* ^2^ = 0.827 (*p* < 0.01)	*R* ^2^ = 0.365 (*p* < 0.01)	*R* ^2^ = 0.253 (*p* = 0.076)

a The topmost value in each box indicates the linear trend (units: % change decade^−1^) along with the associated error of the fit. The middle value indicates the 11 year solar cycle trend component (units: % (100 sfu)^−1^) and associated error, while the *R* ^2^ value of the multilinear regression fit is shown as the lowermost value. All values given to three decimal places.

In order to better understand the causal mechanisms behind these different trends, it is important to examine the long‐term changes in temperature and other chemical species relevant to the respective metal chemistry across the same study period. A similar multilinear regression annual trend analysis was therefore performed for the WACCM‐simulated temperatures at 87, 90, and 95 km (temperatures were determined on a constant altitude level), both stratospheric (10–50 km) and MLT region (75–105 km) O_3_ partial column densities, and CO_2_ and H_2_O column density trends in the MLT, all as a function of latitude band. A summary of these derived trends is presented in Tables 2 and 3. The simulated temperatures indicate a mean decadal global cooling rate of −0.31 K, −0.48 K, and −0.57 K, for the selected altitudes of 87, 90, and 95 km, respectively. It should be noted that a similar global cooling trend is found when considering mean decadal temperature change on constant pressure levels associated with these altitudes, with cooling rates of −0.34 K, −0.33 K, and −0.63 K at constant pressure levels of 0.0024 hPa (≈87 km), 0.0015 hPa (≈90 km), and 0.0005 hPa (≈95 km), respectively. The solar signal is considerably larger than the linear trends, with mean values of +2.17 K, +2.35 K, and +2.16 K, all per 100 sfu for 87, 90, and 95 km, respectively. The stratospheric O_3_ column densities have been decreasing at a mean rate of −2.29% decade^−1^ (mean solar contribution of +0.01%/100 sfu), although this varies greatly with latitude, with the largest decreases in the high southern latitude bands. These changes are related to changing total concentrations of chlorine and bromine species and are largest in spring only, which is not reflected in the annual means analyzed here. The MLT region O_3_ column density exhibits a largely decreasing global trend of −2.49% decade^−1^ (solar: +0.07%/100 sfu). Finally, the CO_2_ mesospheric column densities exhibit increasing global annual linear trends of +0.59% decade^−1^ (solar: +0.02%/100 sfu), while H_2_O generally indicates a decreasing trend at −1.41% decade^−1^ (−0.05%/100 sfu).

**Table 2:** Summary of Derived Linear and 11 Year Solar Cycle Trends for the Temperatures at 87, 90, and 95 km, Within the 50 Year WACCM Time Series, as a Function of Latitude Band^a^

	Linear and 11 Year Solar Cycle Trends in WACCM Metal Layers (Linear Units: K Change Decade^−1^, Solar Trend Units: K (100 sfu)^−1^)		
Latitude Band	*T*—95 km	*T*—90 km	*T*—87 km
60–90°N	−0.474 ± 0.077	−0.323 ± 0.108	−0.149 ± 0.130
	+1.876 ± 0.128	+2.394 ± 0.179	+2.309 ± 0.214
	*R* ^2^ = 0.859 (*p* < 0.01)	*R* ^2^ = 0.800 (*p* < 0.01)	*R* ^2^ = 0.708 (*p* < 0.01)
30–60°N	−0.558 ± 0.066	−0.429 ± 0.071	−0.259 ± 0.076
	+2.186 ± 0.109	+2.469 ± 0.117	+2.337 ± 0.125
	*R* ^2^ = 0.918 (*p* < 0.01)	*R* ^2^ = 0.911 (*p* < 0.01)	*R* ^2^ = 0.875 (*p* < 0.01)
0–30°N	−0.552 ± 0.081	−0.417 ± 0.088	−0.262 ± 0.089
	+2.325 ± 0.134	+2.354 ± 0.145	+2.143 ± 0.147
	*R* ^2^ = 0.893 (*p* < 0.01)	*R* ^2^ = 0.863 (*p* < 0.01)	*R* ^2^ = 0.822 (*p* < 0.01)
0–30°S	−0.563 ± 0.085	−0.425 ± 0.094	−0.269 ± 0.095
	+2.341 ± 0.141	+2.326 ± 0.156	+2.109 ± 0.157
	*R* ^2^ = 0.884 (*p* < 0.01)	*R* ^2^ = 0.845 (*p* < 0.01)	*R* ^2^ = 0.801 (*p* < 0.01)
30–60°S	−0.555 ± 0.085	−0.493 ± 0.095	−0.344 ± 0.094
	+2.172 ± 0.141	+2.294 ± 0.157	+2.069 ± 0.156
	*R* ^2^ = 0.873 (*p* < 0.01)	*R* ^2^ = 0.848 (*p* < 0.01)	*R* ^2^ = 0.806 (*p* < 0.01)
60–90°S	−0.741 ± 0.091	−0.775 ± 0.140	−0.600 ± 0.165
	+2.046 ± 0.150	+2.234 ± 0.231	+2.077 ± 0.273
	*R* ^2^ = 0.875 (*p* < 0.01)	*R* ^2^ = 0.780 (*p* < 0.01)	*R* ^2^ = 0.681 (*p* < 0.01)

a For each box, the values stated are as for Table 1 (linear trend plus error, solar cycle trend plus error, and *R* ^2^ value).

**Table 3:** Summary of Derived Linear and 11 Year Solar Cycle Trends for WACCM Species Relevant to the Metal Chemistries (Partial Column Densities of Stratospheric O_3_ (Between 10 and 50 km), MLT Region O_3_ (75–105 km), MLT H_2_O, and MLT CO_2_), All as a Function of Latitude Band^a^

	Linear and 11 Year Solar Cycle Trends in WACCM Species (Linear Units: % Change Decade^−1^, Solar Trend Units: % (100 sfu)^−1^)			
Latitude Band	Strat O_3_	MLT O_3_	MLT H_2_O	MLT CO_2_
60–90°N	−1.125 ± 0.317	−2.375 ± 0.578	−1.220 ± 0.361	+0.656 ± 0.132
	+0.010 ± 0.013	+0.086 ± 0.065	−0.028 ± 0.386	+0.016 ± 0.539
	*R* ^2^ = 0.483 (*p* < 0.01)	*R* ^2^ = 0.744 (*p* < 0.01)	*R* ^2^ = 0.469 (*p* < 0.01)	*R* ^2^ = 0.686 (*p* < 0.01)
30–60°N	−0.841 ± 0.207	−2.390 ± 0.385	−2.059 ± 0.429	+0.507 ± 0.094
	+0.011 ± 0.014	+0.080 ± 0.074	−0.065 ± 0.185	+0.015 ± 0.574
	*R* ^2^ = 0.582 (*p* < 0.01)	*R* ^2^ = 0.851 (*p* < 0.01)	*R* ^2^ = 0.698 (*p* < 0.01)	*R* ^2^ = 0.738 (*p* < 0.01)
0–30°N	−0.920 ± 0.101	−2.377 ± 0.351	−1.970 ± 0.451	+0.627 ± 0.080
	+0.007 ± 0.025	+0.078 ± 0.076	−0.079 ± 0.144	+0.017 ± 0.489
	*R* ^2^ = 0.810 (*p* < 0.01)	*R* ^2^ = 0.862 (*p* < 0.01)	*R* ^2^ = 0.723 (*p* < 0.01)	*R* ^2^ = 0.834 (*p* < 0.01)
0–30°S	−0.989 ± 0.132	−2.506 ± 0.337	−1.895 ± 0.389	+0.391 ± 0.083
	+0.006 ± 0.029	+0.070 ± 0.086	−0.069 ± 0.163	+0.018 ± 0.478
	*R* ^2^ = 0.768 (*p* < 0.01)	*R* ^2^ = 0.864 (*p* < 0.01)	*R* ^2^ = 0.738 (*p* < 0.01)	*R* ^2^ = 0.801 (*p* < 0.01)
30–60°S	−2.236 ± 0.177	−2.951 ± 0.340	−1.759 ± 0.312	+0.527 ± 0.101
	+0.001 ± 0.119	+0.051 ± 0.108	−0.035 ± 0.356	+0.019 ± 0.504
	*R* ^2^ = 0.884 (*p* < 0.01)	*R* ^2^ = 0.857 (*p* < 0.01)	*R* ^2^ = 0.702 (*p* < 0.01)	*R* ^2^ = 0.759 (*p* < 0.01)
60–90°S	−7.642 ± 0.532	−2.341 ± 0.521	+0.429 ± 0.495	+0.829 ± 0.169
	−0.001 ± 0.303	+0.073 ± 0.092	−0.014 ± 0.840	+0.016 ± 0.603
	*R* ^2^ = 0.901 (*p* < 0.01)	*R* ^2^ = 0.731 (*p* < 0.01)	*R* ^2^ = 0.260 (*p* = 0.068)	*R* ^2^ = 0.606 (*p* < 0.01)

a For each box, the values stated are as for Table 1.

**Figure 3:**
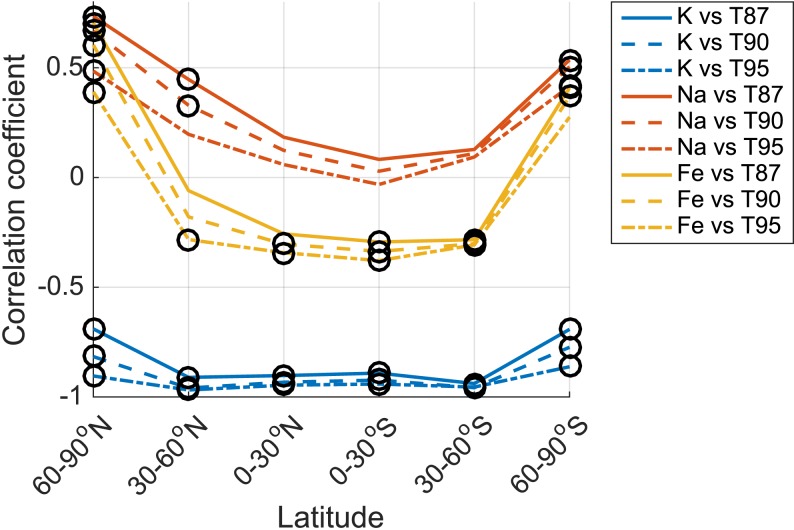
Correlation coefficients for each of the WACCM‐simulated metals versus temperatures across the 50 year period (1955–2005), as a function of latitude. The different metals are represented by different colors: K (blue), Na (red), and Fe (yellow). The different temperatures are indicated by the choice of line: temperature at 87 km (solid), 90 km (dashed), and 95 km (dash‐dotted). All correlation coefficients with a significance value of *p* ≤ 0.05 are circled.

**Figure 4:**
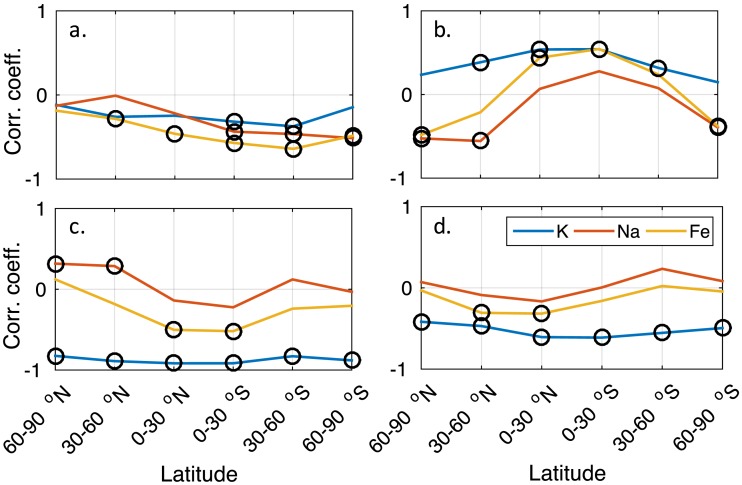
Correlation coefficients for each of the WACCM‐modeled metals versus other relevant chemical species across the 50 year period (1955–2005), all as a function of latitude for MLT region (75–105 km) partial column densities of (a) CO_2_, (b) H_2_O, (c) O_3_, and (d) stratospheric (10–50 km) O_3_. The different metals are represented by different colors: K (blue), Na (red), and Fe (yellow). Correlation coefficients with a significance value of *p* ≤ 0.05 are circled.

A summary of the relationship between the metals and temperature is presented in Figure 3, while the relationship between the metals and the other main chemical species is presented in Figure 4. For both figures, the correlation coefficient between the metal and the variable (either temperature or other chemical species) is shown as a function of latitude, with significant correlations (where the *p* value <0.05) indicated. K column density exhibits a significant and strong negative correlation with temperature at all altitudes, with a mean coefficient value of approximately −0.89. Figure 4 shows that K is the only metal to show a highly significant negative correlation with both stratospheric and mesospheric O_3_ at all latitudes (mean values of approximately −0.53 and −0.88, respectively), while both Na and Fe show weak largely nonsignificant weaker relationships, which depend on latitude. All metals display a weak anticorrelation with CO_2_, with mean global coefficient values of −0.345, −0.472, and −0.489 for K, Na, and Fe, respectively (taking into account only those coefficients which are statistically significant). K is the only metal to show a consistent positive correlation with H_2_O (mean value of +0.361), while both Na and Fe exhibit no overall significant relationship.

### 2.4 Attributing Causal Mechanisms to Explain the Different Long‐Term Behavior

With a long‐term response an order of magnitude larger than that of Na or Fe, it is clear that K is the only metal to exhibit a pronounced response to climate change over the 50 year study period (1955–2005) (see Table 1). An explanation for this differential response is likely found in the different temperature dependency of the K layer chemistry compared to both Na and Fe; this is outlined in *Plane et al*. [2014] and is summarized here. Neutral chemistry dominates on the underside of the layer and for each of K, Na, and Fe, where reactions with O_3_, O_2_, H_2_O, and CO_2_ convert the metal atoms into the major metal reservoirs KHCO_3_, NaHCO_3_, and FeOH [*Plane et al*., 2015]. These reservoir species can be converted back to the neutral metal (hereafter, Mt) via photolysis:

(R1) $$Mt.X+hv\to Mt+X\;\left(\text{where}\;X=OH\;\text{or}\;HCO_{3}\right)$$

or via reaction with H:

(R2) MtX+H→Mt+HX

For Na, (R2) has an activation energy of approximately 9.7 kJ mol^−1^ [*Cox et al*., 2001]. Hence, this reaction becomes very slow during the cold temperatures of the summertime mesopause, resulting in a buildup of NaHCO_3_ relative to atomic Na. Similarly, the analogous reaction of FeOH also has a relatively small activation energy of 10.5 kJ mol^−1^ [*Plane et al*., 2015]. In contrast, the activation energy for the KHCO_3_ + H reaction is so large (~34 kJ mol^−1^) that the reaction is too slow to influence the K layer even at the warmer temperatures of the wintertime MLT. The only way that KHCO_3_ is converted back to atomic K is via photolysis. As none of the reactions which convert K to KHCO_3_ has significant temperature dependencies, the overall result is that the neutral K chemistry on the underside of the layer is essentially temperature independent.

On the topside of the metal layers, ion chemistry dominates, and Mt^+^ ions are formed as a result of both photoionization and charge transfer with ambient ions ((R3)–(R5)):

(R3) $$Mt+hv\to Mt^{+}+e^{-}$$

(R4) $$Mt+NO^{+}\to Mt^{+}+\text{NO}$$

(R5) $$Mt+{O_{2}}^{+}\to Mt^{+}+O_{2}$$

Note that charge transfer dominates over photoionization [*Plane et al*., 2015]. The Mt^+^ ions may form clusters with an available ligand or in the case of Fe^+^ react with O_3_ to form FeO^+^, before the resulting molecular ions undergo dissociative recombination with electrons to yield the neutral Mt atom:

(R6) $$Mt^{+}.X+e^{-}\to Mt+X\;\left(\text{where}\;X=N_{2},\;O,\;CO_{2}\;\text{or}\;H_{2}O\right)$$

In contrast to Na and Fe, the relatively large singly charged K^+^ ion forms only weakly bound clusters with low binding energies of <20 kJ mol^−1^ [*Plane et al*., 2014]. These clusters can only form at the very cold temperatures of the summertime MLT, which then converts the reservoir K^+^ into K, leading to the K summertime maximum not seen in the other metals.

The modeled K layer column density shows an increasing trend at all latitudes (Table 1) and exhibits a significant anticorrelation with temperature (Figure 3). This is consistent with the increasing rate of conversion of K^+^ to K at lower temperatures, together with the temperature‐independent neutral chemistry. Both the Na and Fe column densities exhibit long‐term responses an order of magnitude smaller than that of K and a strong latitude dependence of the correlation of metal atom density with temperature. These responses are, in part, consistent with the temperature dependence of reaction (R2); as temperatures decrease a larger fraction of the metal is converted to NaHCO_3_ or FeOH. The varying response of Na and Fe is also caused by the latitude‐dependent behavior of CO_2_, H_2_O, and O_3_ and their corresponding relationship with temperature (Table S1), because these species are involved in the reactions which form the reservoir species NaHCO_3_ and FeOH. Of these, Fe exhibits the greatest latitude‐dependent relationship with temperature of the three metals (see Figure 3); this is likely due to the lower peak layer altitude (~85–87 km) of the Fe layer compared to the Na and K layers (90–93 km) and the resulting altitude‐dependent interplay between the metal chemistry, relevant species, and temperature.

### 2.5 Long‐Term Changes in the Modeled Metal Layer Centroid Altitude and Width

**Figure 5:**
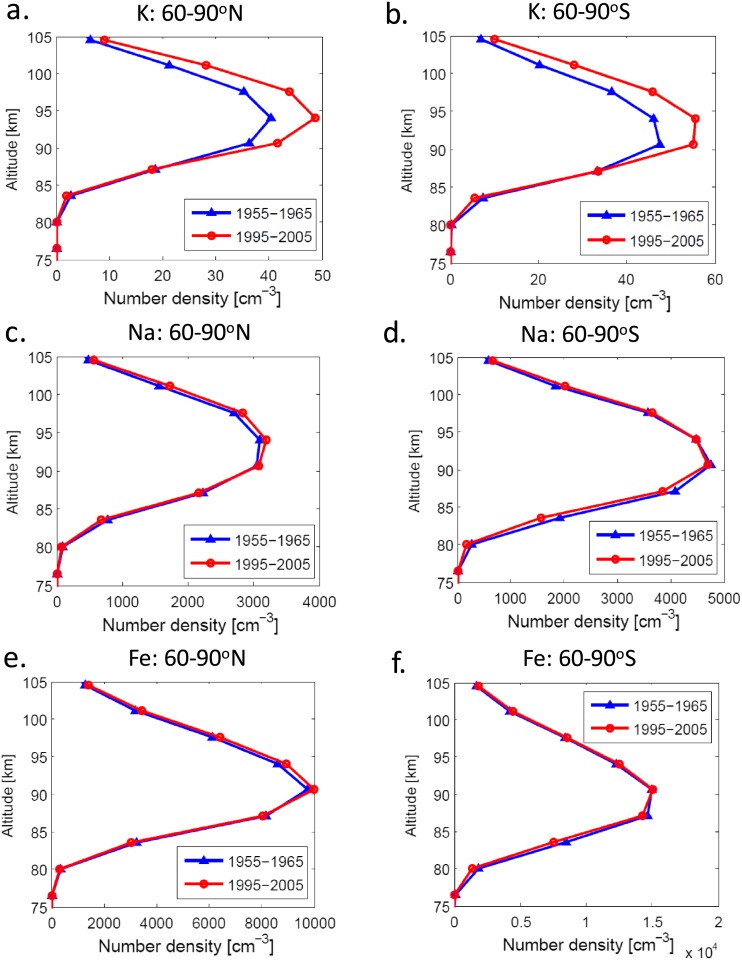
Comparison of the long‐term response of (a and b) K, (c and d) Na, and (e and f) Fe across a 50 year period (1955–2005). All profiles show the respective mean metal number density across 1955–1965 (blue profiles) versus 1995–2005 (red profiles), as a function of altitude. The left‐hand column indicates zonal mean profiles for 60–90°N, and the right‐hand column indicates zonal mean profiles for 60–90°S.

Figure 5 summarizes the long‐term changes in the shape of the modeled K, Na, and Fe metal layers at fixed‐altitude levels across 1955–2005. Overall, the changes in centroid altitude for all metals are very small, typically varying between 0 and +0.02% yr^−1^, which equates to a maximum change of <1% across the 50 year period. This is in good agreement with *Clemesha et al*. [2004], who found a negligible long‐term trend in the vertical distribution of the Na layer measured at São José dos Campos (23°S, 46°W) from 1971 to 2001. Similarly, the root‐mean‐square (RMS) widths of the metal layers exhibit no overall net change with a maximum change also <1% from 1955 to 2005.

## 3 The Solar Cycle Response of the Na and K Layers

### 3.1 The Observed and Modeled Na and K Metal Layers

The ~11 year solar cycle (hereafter referred to as SC) describes the periodic changes in sun spot and flare activity on the surface of the Sun, and it is an important source of variation within the whole atmosphere [e.g., see *Haigh*, 1996; *Shindell et al*., 1999; *Laštovička*, 2005]. SC impacts are predominantly caused by changes in temperature and rates of photoionization and photodissociation.

A robust solar cycle trend analysis would include data which cover at least two solar cycles, i.e., a data set of 25–30 years. However, in this work we have had to use Na and K dayglow emissions measured by the OSIRIS (Optical Spectrograph and InfraRed Imaging System) instrument on the Odin satellite, which are converted into absolute atomic density profiles using the retrieval schemes first described in *Gumbel et al*. [2007] and *Dawkins et al*. [2014], respectively. These near‐global retrieved data sets extend from 2004 to mid‐2013 and span portions of solar cycles 23 and 24. Both the relatively short time series and the fact that the solar cycle 23–24 transition was unusually quiet [e.g., *Agee et al*., 2010] present obvious limitations to studying the impact of the solar cycle on the metal layers. Nevertheless, the observations can be used to test the model prediction of a different SC response of the K and Na layers.

In this work, the solar 10.7 cm radio flux is used as a proxy for solar cycle activity, in addition to temperatures at 87, 90, and 95 km derived from the SABER (Sounding of the Atmosphere using Broadband Emission Radiometry) instrument on‐board the NASA TIMED (Thermosphere Ionosphere Mesosphere Energetics and Dynamics) mission satellite. Although temperature is dependent on many factors, including dynamical effects and variations in local chemical heating, there is a pronounced SC effect. *Forbes et al*. [2014] demonstrated the temperature variability associated with the SC using data from both WACCM and SABER. For fixed altitudes, they found that the SABER data showed a temperature sensitivity of 1–2 K/100 sfu below 70 km, compared to 3 K/100 sfu for WACCM. Above this altitude, they found that the temperature of both data sets increased: SABER showed a sensitivity of 4–6 K/100 sfu at 95 km in the range ±50° latitude, which increased to approximately 10–14 K/100 sfu at higher latitudes. WACCM also showed an increased sensitivity with a similar qualitative response, but the overall temperature sensitivity was weaker by a factor of 2 compared to the SABER observations.

Table 4 presents the correlations between the observed (OSIRIS) and modeled (WACCM) K and Na layers and the various SC proxies. Only model data between 82°N and 82°S have been used in order to reflect the coverage extent of the satellite instrument. Additionally, point source data from the K lidar at Kühlungsborn (54°N, 12°E) are also included. All time series were normalized to the respective monthly means, and the correlation analyses were performed on the resulting monthly data sets.

**Table 4:** Correlation Analyses Between the Observed and Modeled K and Na Global Mean Column Densities and the Solar *F*_10.7_ Flux (*F*_10.7_) and Global Mean SABER Temperatures at 87 km (T87), 90 km (T90), and 95 km (T95), Respectively^a^

	K			Na	
	OSIRIS	KBORN	WACCM	OSIRIS	WACCM
*F*_10.7_	**+0.217 (*p* < 0.05)**	−0.180 (*p* = 0.102)	**−0.693 (*p* < 0.01)**	+0.194 (*p* = 0.081)	**+0.193 (*p* < 0.05)**
T87	−0.047 (*p* = 0.622)	−0.111 (*p* = 0.316)	**−0.208 (*p* < 0.05)**	+0.178 (*p* = 0.110)	+0.101 (*p* = 0.280)
T90	−0.055 (*p* = 0.564)	−0.204 (*p* = 0.063)	**−0.215 (*p* < 0.05)**	+0.103 (*p* = 0.359)	+0.097 (*p* = 0.302)
T95	+0.016 (*p* = 0.864)	+0.128 (*p* = 0.247)	**−0.260 (*p* < 0.01)**	+0.048 (*p* = 0.666)	+0.134 (*p* = 0.152)

a All data cover the period 2004–2013. Both the OSIRIS and WACCM metal data sets represent the global mean column density between 82°N and 82°S, to reflect the coverage of the satellite. The lidar K data set (KBORN) is from the Kühlungsborn lidar (54°N, 12°E) and thus does not represent a global mean. The confidence value of each correlation coefficient is indicated in brackets. Results in bold indicate a confidence level of ≥95%.

The WACCM data set exhibits a weak, though statistically significant, anticorrelation between K and the temperatures at both 87 km (−0.208, *p* < 0.05) and 90 km (−0.215, *p* < 0.05). The mean correlation coefficient between OSIRIS K and the temperature at both 87 and 90 km is approximately −0.051, compared to a mean value of −0.212 for WACCM K and −0.158 for the lidar K data. While both the OSIRIS and lidar data sets indicate no significant correlation between K and temperature at 95 km (OSIRIS: +0.016, *p* = 0.864; lidar: +0.128, *p* = 0.247), WACCM indicates a significant anticorrelation (−0.260, *p* < 0.01). The data sets vary in their response to the *F*_10.7_ index: both the lidar and WACCM data sets exhibit an anticorrelation of various strengths (−0.180, *p* = 0.102, and −0.693, *p* < 0.01, respectively) in contrast to the significant weak positive correlation between OSIRIS and this index (+0.217, *p* < 0.05).

The correlation analyses for Na are quite different from those for K. Both the OSIRIS and WACCM Na data sets exhibit a very similar, weak positive correlation with the *F*_10.7_ index, which is significant for WACCM only (WACCM: +0.193, *p* < 0.05; OSIRIS: +0.194, *p* = 0.081). In contrast to K, neither the OSIRIS nor WACCM Na data sets exhibit any overall significant trend with temperature at any of the three altitudes (i.e., all exhibit nonstatistically significant weak positive correlations between +0.048 and +0.178).

### 3.2 Explaining the 11 Year Solar Cycle Response of Observed and Modeled Na and K

The impact of the solar cycle can be divided into two categories: changes in photoionization and photodissociation rates and changes in temperature. Both changes in photoionization and photodissociation affect the K and Na metal layers equally. During solar maximum conditions, an increased rate of photoionization occurs resulting in an enhanced concentration of metal ions partly through reaction (R3) but more importantly via (R4) and (R5) due to an associated increase in the amount of ambient *E* region ions and the corresponding increase in the rate of charge transfer reactions. The result is that a greater proportion of K and Na are present as ions, rather than neutral atoms during solar maximum. Changes in the rate of photodissociation may also be important for the metal chemistry of the layer underside. The concentrations of atomic O and H are also higher during solar maximum, and these species convert metal compounds back to the neutral atoms, e.g.,

(R7) $$MtO+O\to Mt+O_{2}$$

(R8) $$\text{MtHC}O_{3}+H\to Mt+H_{2}CO_{3}$$

The critical factor in explaining the difference in the apparent solar cycle response lies in (R8), which is only relevant for the Na chemistry; the activation energy for the analogous K reaction is too high to be viable at temperatures within the MLT (see above). Thus, during solar maximum, enhanced temperatures and H atom concentrations increase the rate of (R8), leading to an enhancement of Na. There is no equivalent effect in K. The second temperature‐related dependence occurs as a result of the differences in the ion chemistry of both species as discussed in section 2.4.

**Figure 6:**
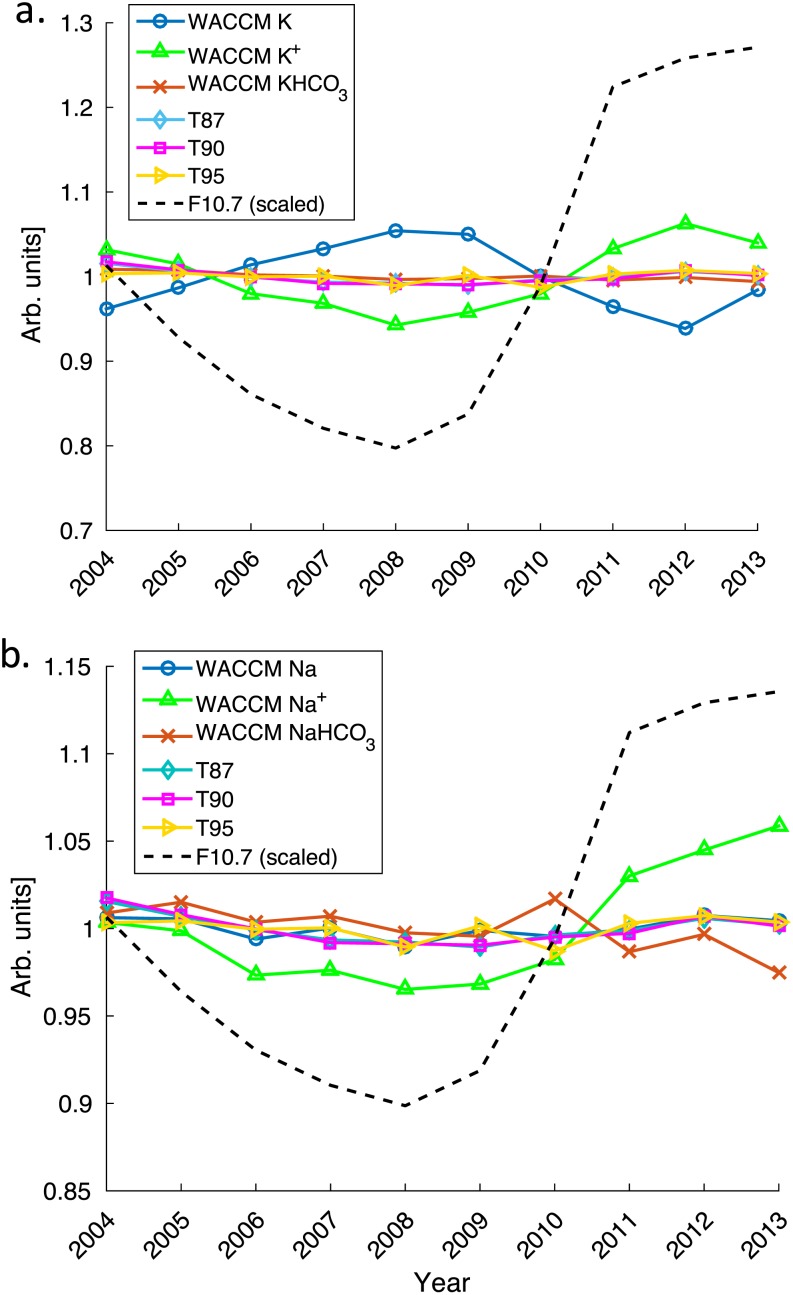
Response of the WACCM‐modeled (a) K and (b) Na layer to the 11 year solar cycle. All results have been normalized (divided by their means) to allow for easy comparison. The solar flux (represented by the *F*_10.7_ radio flux) is shown as the dashed line. For both metals, the neutral metal column density (between 75 and 105 km) is shown as the solid blue line (with circles), the metal ion column density as green line (with triangles), and the metal bicarbonate as the red line (with crosses). SABER temperatures at 87, 90, and 95 km are shown as the cyan (diamond), magenta (square), and yellow (triangles) lines, respectively.

The overall response of the K layer is an anticorrelation with the solar cycle; the reduced rates of photoionization and lower temperatures associated with solar minimum promote enhanced K concentrations. Although photodissociation of K reservoir species is a source of neutral K via (R1) (and thus reducing it will reduce the K), it does not appear to be as important here. While both the OSIRIS and WACCM K correlations support an anticorrelation between the *F*_10.7_ flux and temperature, care must be taken in the interpretation, as the changes in SABER temperature at 87, 90, and 95 km are not linearly related to the solar flux alone. A schematic overview of this relationship is provided in Figure 6.

In the case of Na, the temperature dependence of reaction (R8) results in an enhancement of the neutral Na layer during warmer solar maximum conditions. However, this is offset by the increased rate of photoionization during solar maximum. These competing effects result in little overall solar cycle response, as shown in the nonsignificant near‐zero OSIRIS and WACCM Na versus *F*_10.7_ correlation coefficients.

## 4 Summary and Conclusions

There is increasing interest in the response of the Earth's atmosphere to both solar cycle variations and longer‐term changes as a result of anthropogenic climate change. To date the majority of research has focused on the impacts of anthropogenic climate change within the lower atmosphere. However, there is increasing evidence that changes in greenhouse gas concentrations are impacting all parts of the atmosphere, with the middle and upper atmosphere particularly sensitive to such changes [*Plane et al*., 2015, and references therein].

In this study we have used the WACCM model to assess the long‐term responses of the K, Na, and Fe metal layers within the MLT region. K is the only layer which shows significant long‐term changes in column density, caused by the more efficient conversion of K^+^ into K at lower temperatures and the relative temperature independence of the neutral chemistry which partitions K into the reservoir species KHCO_3_. In contrast, both Na and Fe have temperature‐dependent neutral chemistries, so that increased conversion of their ions into neutral atoms at lower temperatures is offset by the greater stability of the neutral reservoirs NaHCO_3_ and FeOH. The long‐term trends in the Na and Fe column densities are much less significant; they are much more sensitive to the subtle interaction of latitude‐specific changes in temperature and O_3_, CO_2_, and H_2_O within the MLT region. No significant long‐term trends are evident in the centroid height and RMS widths of any of the modeled metal layers, which is in good agreement with the findings of *Clemesha et al*. [2004] in the case of the Na layer.

The solar cycle is one of the fundamental sources of natural variation within the Earth's atmosphere, and its influence must be understood in order to determine underlying longer‐term trends. The results presented here should be treated with some caution because the observations of Na and K do not cover even one full solar cycle but a 10 year period from 2004 to 2013 (ideally, a trend analysis requires data extending over at least two solar cycles for robust conclusions to be drawn). In addition, the latter half of solar cycle 23 and the current cycle 24 are unusually quiet, which makes any solar cycle response harder to detect.

The OSIRIS and WACCM Na and K data sets were examined in response to the solar *F*_10.7_ index and temperatures from the TIMED/SABER instrument. Both the observed and modeled data sets indicate that the Na and K layers exhibit different responses to the solar cycle. K shows a highly significant anticorrelation (reduced neutral K during solar maximum and enhanced K during solar minimum). Meanwhile, the Na layer displays a much less significant solar cycle response, due to the competing influences of temperature and photoionization on its chemistry.

The trends in the OSIRIS observations and WACCM data agree qualitatively but not quantitatively. The OSIRIS metal data sets show considerably more natural variability than seen in WACCM, which is likely to account for many of the differences in the absolute strengths of the trends and the correlation analyses. In addition, the WACCM solar cycle temperature response is not as pronounced as seen in the SABER temperature data; this too will contribute to the quantitative differences between the OSIRIS and WACCM trends.

Overall, the results presented here demonstrate that the unusual behavior of K compared to Na and Fe is present at diurnal [*Feng et al*., 2015], seasonal [*Dawkins et al*., 2015], and longer timescales (this study). The model predicts that K is the only one of these three metals that will provide a sensitive indicator of long‐term changes in the MLT region.

## Supporting information



Supporting Information S1

Figure S1

Table S1
