# Supplementary material for: Solar cycle response and long‐term trends in the mesospheric metal layers
Source: J Geophys Res Space Phys. 2016 Jul 27;121(7):7153–65. doi: 10.1002/2016JA022522 (PMC6680104; doi:10.1002/2016JA022522)
Supplement: Supplementary file 1 — Supporting Information S1 [file JGRA-121-7153-s001.doc]

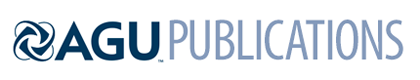


*Journal of Geophysical Research*

Supporting Information for

**Solar cycle response and long-term trends in the mesospheric metal layers**

E.C.M. Dawkins^1,2,5^, J.M.C. Plane^*1^, M.P. Chipperfield^2^, W. Feng^1,2^ , D.R. Marsh^3^, J. Höffner^4^ and D. Janches^5^

1. School of Chemistry, University of Leeds, UK

2. School of Earth and Environment, University of Leeds, UK

3. National Center for Atmospheric Research, Boulder, Colorado, USA

4. Leibniz-Institute for Atmospheric Physics, Kühlungsborn, DE

5. NASA Goddard Space Flight Center, Maryland, USA

* Corresponding author (j.m.c.plane@leeds.ac.uk)

**Contents of this file**

Text S1

Figure S1

Table S1

**Introduction**

The following figures and table provide supporting information to the main manuscript.

Text S1.

The Fast-Fourier transform spectral analyses of the periodicities of the WACCM modeled metal layers are presented in Figure S1 and demonstrate that all the most significant sources of variation are adequately accounted for in the least-squares multi-linear fitting routine. Table S1 presents the correlation coefficient, r (and associated significance) between the WACCM modeled temperature at three heights (87, 90 and 95 km) and other variables (stratospheric O_3_ integrated over 10-50 km, MLT O_3_ integrated over 75-105 km, MLT CO_2_ and MLT H_2_O), all of which are relevant to the metal layer chemistry.

**Figure S1.** Fast-Fourier transform spectral analysis of periodicities of K, Na and Fe WACCM model output, all for 60-90^o^N. Each inset plot presents an expanded y-axis for clarity. [Units of frequency: yr^-1^].

**Table S1.** Correlations between temperature (at 87, 90, and 95 km) and stratospheric and mesospheric O_3_, and MLT region CO_2_ and H_2_O, examined across a 50-year period (1955-2005). All simulated by WACCM.

|  | **Latitude band** | | | | | |
| --- | --- | --- | --- | --- | --- | --- |
|  |  | | | | | |
|  | 60-90^o^N | 30-60^o^N | 0-30^o^N | 0-30^o^S | 30-60^o^S | 60-90^o^S |
| MLT CO_2_ | |  |  |  |  |  |
| T95 | *+0.011* | *+0.231* | *+0.143* | *+0.179* | *+0.256* | *-0.180* |
| T90 | *+0.016* | +**0.294** | *+0.219* | *+0.241* | +**0.280** | **-0.349** |
| T87 | *-0.028* | +**0.312** | *+0.273* | +**0.291** | +**0.281** | **-0.415** |
|  | | | | | | |
| MLT H_2_O | |  |  |  |  |  |
| T95 | *-0.219* | **-0.312** | **-0.400** | **-0.391** | *-0.188* | *-0.220* |
| T90 | **-0.422** | **-0.466** | **-0.480** | **-0.460** | **-0.276** | **-0.427** |
| T87 | **-0.534** | **-0.595** | **-0.553** | **-0.527** | **-0.380** | **-0.532** |
|  | | | | | | |
| MLT O_3_ | |  |  |  |  |  |
| T95 | **+0.674** | **+0.878** | **+0.857** | **+0.860** | **+0.829** | **+0.775** |
| T90 | **+0.596** | **+0.883** | **+0.827** | **+0.817** | **+0.788** | **+0.630** |
| T87 | **+0.478** | **+0.845** | **+0.779** | **+0.760** | **+0.718** | **+0.506** |
|  | | | | | | |
| Strat. O_3_ | |  |  |  |  |  |
| T95 | **+0.280** | **+0.555** | **+0.587** | **+0.688** | **+0.623** | **+0.581** |
| T90 | *+0.173* | **+0.452** | **+0.493** | **+0.627** | **+0.569** | **+0.450** |
| T87 | *+0.082* | **+0.332** | **+0.392** | **+0.556** | **+0.501** | **+0.315** |
