# Supplementary figures and images for: Solar cycle response and long‐term trends in the mesospheric metal layers
Source: J Geophys Res Space Phys. 2016 Jul 27;121(7):7153–65. doi: 10.1002/2016JA022522 (PMC6680104; doi:10.1002/2016JA022522)

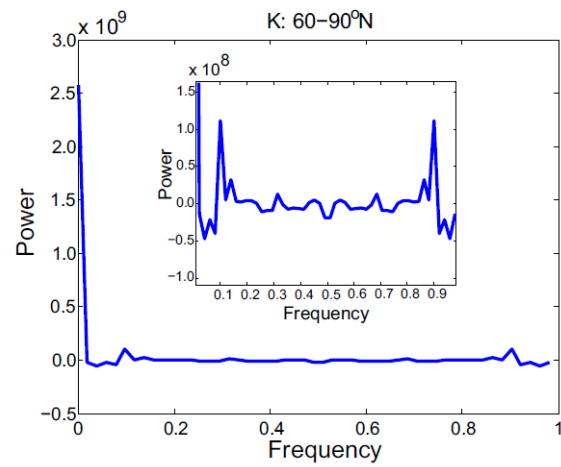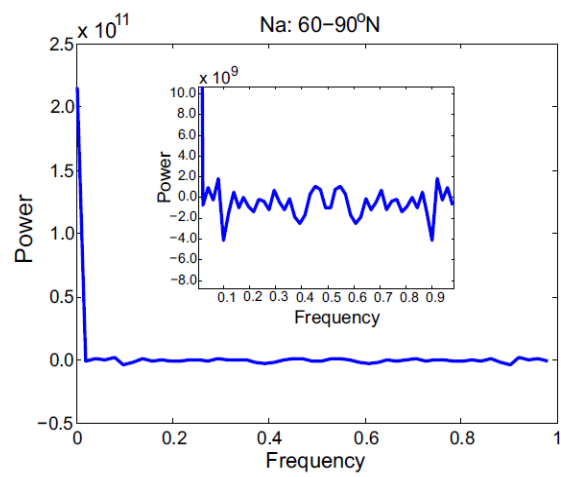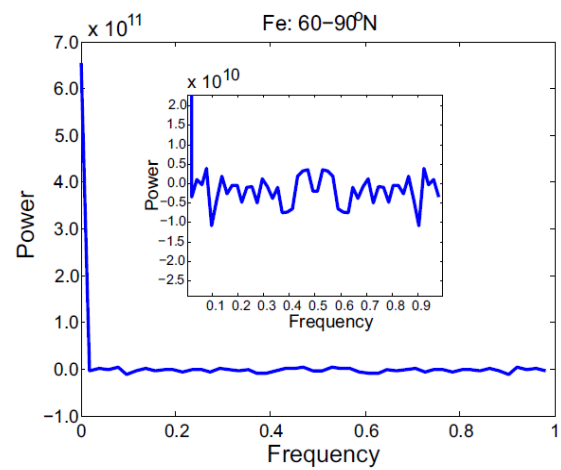

Supplement: Supplementary file 2 — Figure S1 [file JGRA-121-7153-s002.pdf]
