# Supplementary material for: Solar cycle response and long‐term trends in the mesospheric metal layers
Source: J Geophys Res Space Phys. 2016 Jul 27;121(7):7153–65. doi: 10.1002/2016JA022522 (PMC6680104; doi:10.1002/2016JA022522)
Supplement: Supplementary file 3 — Table S1 [file JGRA-121-7153-s003.docx]

**Table S1.** Correlations between temperature (at 87, 90, and 95 km) and stratospheric and mesospheric O_3_, and MLT region CO_2_ and H_2_O, examined across a 50-year period (1955-2005). All simulated by WACCM.

|  | **Latitude band** | | | | | |
| --- | --- | --- | --- | --- | --- | --- |
|  |  | | | | | |
|  | 60-90^o^N | 30-60^o^N | 0-30^o^N | 0-30^o^S | 30-60^o^S | 60-90^o^S |
| MLT CO_2_ | |  |  |  |  |  |
| T95 | *+0.011* | *+0.231* | *+0.143* | *+0.179* | *+0.256* | *-0.180* |
| T90 | *+0.016* | +**0.294** | *+0.219* | *+0.241* | +**0.280** | **-0.349** |
| T87 | *-0.028* | +**0.312** | *+0.273* | +**0.291** | +**0.281** | **-0.415** |
|  | | | | | | |
| MLT H_2_O | |  |  |  |  |  |
| T95 | *-0.219* | **-0.312** | **-0.400** | **-0.391** | *-0.188* | *-0.220* |
| T90 | **-0.422** | **-0.466** | **-0.480** | **-0.460** | **-0.276** | **-0.427** |
| T87 | **-0.534** | **-0.595** | **-0.553** | **-0.527** | **-0.380** | **-0.532** |
|  | | | | | | |
| MLT O_3_ | |  |  |  |  |  |
| T95 | **+0.674** | **+0.878** | **+0.857** | **+0.860** | **+0.829** | **+0.775** |
| T90 | **+0.596** | **+0.883** | **+0.827** | **+0.817** | **+0.788** | **+0.630** |
| T87 | **+0.478** | **+0.845** | **+0.779** | **+0.760** | **+0.718** | **+0.506** |
|  | | | | | | |
| Strat. O_3_ | |  |  |  |  |  |
| T95 | **+0.280** | **+0.555** | **+0.587** | **+0.688** | **+0.623** | **+0.581** |
| T90 | *+0.173* | **+0.452** | **+0.493** | **+0.627** | **+0.569** | **+0.450** |
| T87 | *+0.082* | **+0.332** | **+0.392** | **+0.556** | **+0.501** | **+0.315** |
